# Biomechanical comparison of the femoral neck system and the dynamic hip screw in basicervical femoral neck fractures

**DOI:** 10.1038/s41598-022-11914-1

**Published:** 2022-05-12

**Authors:** Jun-Ki Moon, Jung Il Lee, Kyu-Tae Hwang, Jae-Hyuk Yang, Ye-Soo Park, Ki-Chul Park

**Affiliations:** 1 Department of Orthopaedic Surgery, Hanyang University Guri Hospital, Gyomoon-dong 249-1, Guri City, Gyunggi-do 471-701 South Korea; 2 Department of Orthopedic Surgery, Hanyang University Hospital, Seoul, South Korea; 3 Department of Orthopaedic Surgery, Chung-Ang University Gwangmyeong Hospital, Gwangmyeong, South Korea

**Keywords:** Medical research, Physics

## Abstract

The purpose of this study was to compare the fixation stability of proximal fragments and the mechanical characteristics in proximal femur models of basicervical femoral neck fracture fixed by the femoral neck system (FNS) versus the dynamic hip screw. The mean axial stiffness was 234 ± 35 N/mm in the FNS group and 253 ± 42 N/mm in the DHS group, showing no significant difference (p = 0.654). Mean values for x-axis rotation, y-axis rotation, and z-axis rotation after cycle load were 2.2 ± 0.5°, 6.5 ± 1.5°, and 2.5 ± 0.6°, respectively, in the FNS group and 2.5 ± 0.7°, 5.8 ± 2.1°, and 2.2 ± 0.9°, respectively, in the DHS group, showing no significant differences (p = 0.324, p = 0.245, and p = 0.312, respectively). The mean values of cranial and axial migration of screws within the femoral head were 1.5 ± 0.3 and 2.1 ± 0.2 mm, respectively, in the FNS group and 1.2 ± 0.3 and 2.4 ± 0.3 mm, respectively, in the DHS group, showing no significant differences (p = 0.425 and p = 0.625, respectively). The average failure load at vertical load was 1342 ± 201 N in the FNS group and 1450 ± 196 N in the DHS group, showing no significant difference (p = 0.452). FNS fixation might provide biomechanical stability comparable to that of DHS for treating displaced basicervical femoral neck fractures in young adults.

## Introduction

Hip fracture is an important and debilitating condition in older patients, and the global incidence of hip fracture is rising^1^. The most common site affected is the femoral neck, although intertrochanteric fractures are similar in incidence in older patients^2^. Current implant selections for femoral neck fractures are an important and controversial topic with regard to the extent of displacement, fracture configuration, age of patients, and other factors. In elderly patients with displaced femoral neck fractures, arthroplasty tends to be preferred, whereas internal fixation is preferred for young adults^3,4^. Anatomic reduction and rigid internal fixation are essentials for achieving treatment goals in young adults as they allow preservation of the femoral head while minimizing rates of osteonecrosis^5^.

Basicervical femoral neck fracture is an uncommon fracture type, which is located at the junction between the femoral neck and intertrochanteric region^6^. It is rare, with an incidence of 1.8% to 7.6% among hip fractures^7,8^. Because of its anatomical nature, basicervical femoral neck fracture remains a controversial fracture type, being regarded as an intracapsular or extracapsular fracture^9^. Internal fixation using a dynamic hip screw (DHS) or an intramedullary nail has been performed as a treatment method for these fractures^9,10^. Some biomechanical studies reported that DHS and intramedullary nails achieved higher fixation strengths compared to cannulated screws in basicervical femoral neck fracture^9,11^.

The femoral neck system (FNS), which is a minimally invasive implant recently developed for the treatment of femoral neck fractures, including basilar, transcervical, and subcapital fractures, combines the advantages of angular stability with the minimally invasive surgical technique^12^. A previous biomechanical study conducted by Stoffel et al.^12^ reported that FNS showed biomechanical stability comparable to the DHS and superior to cannulated screws in unstable Pauwels III femoral neck fractures. Although FNS fixation has also been indicated for basicervical femoral neck fracture, no study has compared the biomechanical properties of FNS and DHS fixation in basicervical femoral neck fracture. A recent report concluded that the basicervical fracture should be treated as an extracapsular fracture, such as the trochanteric fracture, which is contraindicated for FNS fixation^13^. Therefore, it is important to document the biomechanical properties of FNS fixation by comparing it with DHS in basicervical femoral neck fractures.

The purpose of the present study was to compare FNS and DHS fixation with regard to 1) the fixation stability of the proximal fragment, 2) their mechanical characteristics in proximal femur models of basicervical neck fracture, 3) whether FNS fixation could achieve comparable clinical outcomes with DHS fixation based on a biomechanical test.

## Results

### Axial stiffness

The mean value of axial stiffness during vertical loads increasing by 500 N was 234 ± 41 N/mm in the FNS group and 253 ± 42 N/mm in the DHS group. There was no significant difference in axial stiffness between the two groups (p = 0.654).

### Changes after cyclic load

The mean value for varus collapse (x-axis rotation) after 10,000 cycles was 2.2 ± 0.5° in the FNS group and 2.5 ± 0.7° in the DHS group, showing no significant difference (p = 0.324). There were no significant differences between the FNS and DHS groups in the mean values for y-axis rotation (6.5 ± 1.5° vs. 5.8 ± 2.1°, p = 0.245) and z-axis rotation (2.5 ± 0.6° vs. 2.2 ± 0.9°, p = 0.312). The mean values for cranial and axial screw migration within the femoral head were 1.5 ± 0.3 mm and 2.1 ± 0.2 mm, respectively, in the FNS group and 1.2 ± 0.3 mm and 2.4 ± 0.3 mm, respectively, in the DHS group. There were no significant differences in cranial and axial screw migration between the groups (p = 0.425 and p = 0.625, respectively).

### Construct failure

Construct failure occurred in 12 cases as excessive displacement of > 15 mm of the proximal fragment and in 5 cases as peri-implant fracture. In three cases, a sudden decrease in load resistance was observed at the load–displacement curve before any of the other failure criteria was fulfilled.

**Figure 1:**
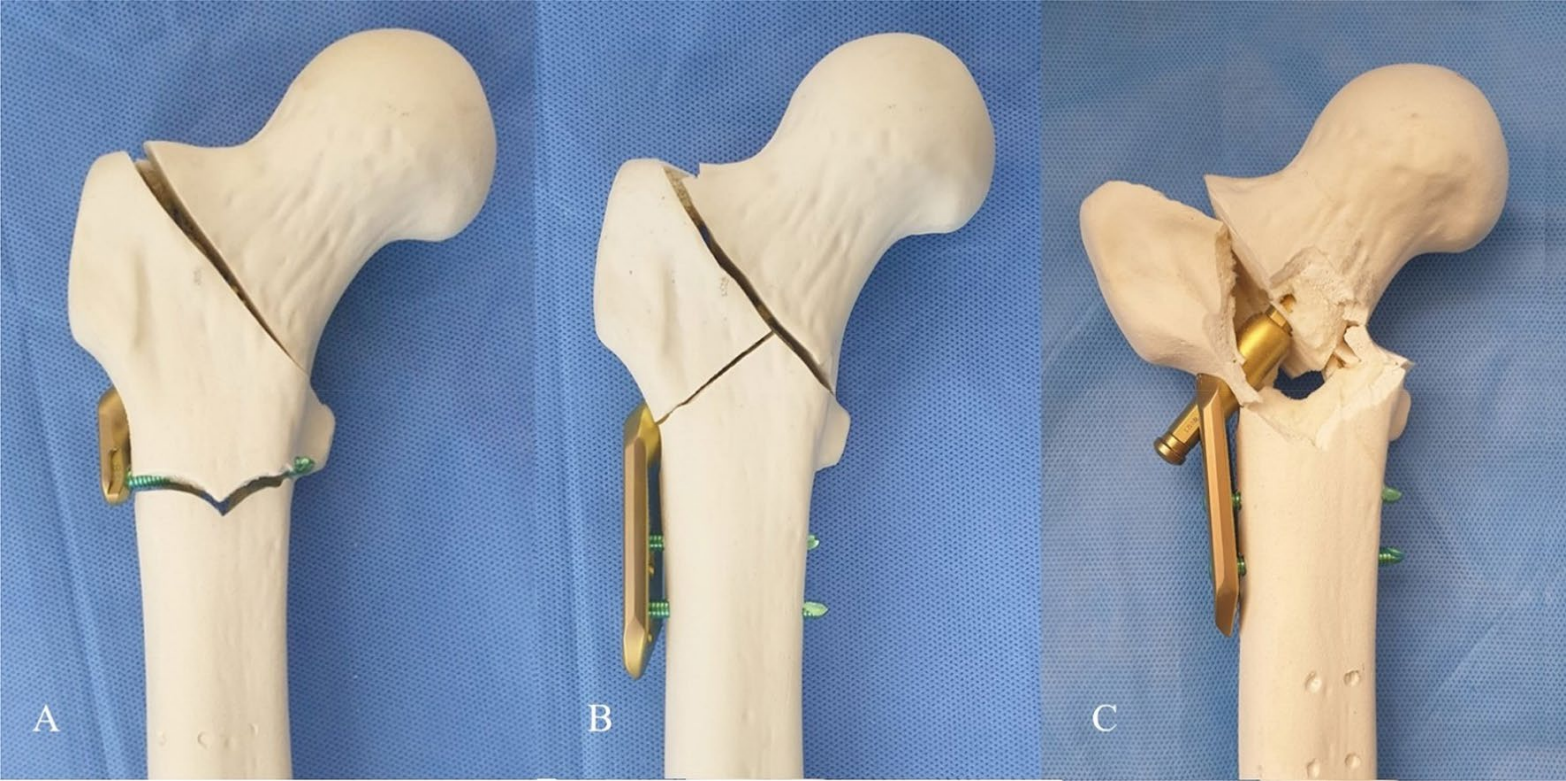
Specimens showing different locations of construct failure including (**a**) peri-implant fracture distal to screw hole in FNS group, (**b**) lateral wall fracture around lag screw in DHS group, and (**c**) displaced lateral wall fracture with varus collapse and lag screw pull-out in DHS group.

Representative photographs of construct failure are shown in Fig. 1. The distribution of failure types in each group is presented in Table 1. There was no significant difference in the distribution of failure type between the two groups (p = 0.666). The average failure load nuybwas 1342 ± 201 N in the FNS group and 1450 ± 196 N in the DHS group, showing no significant difference (p = 0.452).

**Table 1:** Types of construct failure.

	Type of construct failure	No.
FNS group	Peri-implant fracture (locking screw hole)	3
	Displacement of the fragment more than 15 mm	6
	Sudden decrease of the load resistance	1
DHS group	Peri-implant fracture (lateral wall fracture around lag screw)	2
	Displacement of the fragment more than 15 mm	6
	Sudden decrease of the load resistance	2

## Discussion

The present study compared FNS and DHS fixation with respect to the fixation stability of the proximal fragment and the mechanical properties in proximal femur models of displaced basicervical neck fracture. Biomechanical testing showed no significant differences in mechanical properties, including mean axial stiffness, migration of the proximal fragment and screw tip within the femoral head after cyclic loads, and failure load. To the best of my knowledge, the present study is the first to compare fixation stability between FNS and DHS fixation for the treatment of basicervical femoral neck fracture.

Basicervical femoral neck fractures are rare, and are treated differently compared to other intracapsular femoral neck fractures^9^. Young adults tend to have more vertically oriented basicervical fractures from high-energy mechanisms in which an axial load is applied to an abducted knee^14–16^. In contrast, elderly patients who have poorer bone quality more frequently have transcervical or subcapital femoral neck fractures from low-energy mechanisms^17^. Therefore, basicervical femoral neck fractures might be treated differently from other types of femoral neck fracture that frequently occur in elderly patients. In the present study, composite femurs coated with a thin cortical layer and filled with solid density polyurethane foam were used to simulate these situations in young adults.

Recently, FNS was introduced with the intention of combining the increased fracture fixation properties of DHS systems and the minimally invasive approach^18^. A minimized footprint on the bone and a reduced length of incision during FNS fixation might be advantageous clinically compared with DHS fixation. However, it is unclear whether FNS provides comparable fixation stability when treating a displaced basicervical femoral neck fracture, which reportedly should be treated as an extracapsular fracture such as an intertrochanteric fracture. FNS fixation is contraindicated for intertrochanteric fracture of the femur because of the high failure rate based on early pilot studies conducted in the medical device industry. Basicervical femoral neck fractures have been reported to have greater biomechanical instability than stable intertrochanteric fractures due to greater fracture angle and subsequent greater varus moment, which may lead to poor functional outcome^9,19,20^. Therefore, basicervical femoral neck fractures may also have increased failure rates when treated with FNS fixation. However, the present study showed that FNS fixation provided comparable fracture stability in terms of rotational change of the proximal fragment and bolt tip migration within the femoral head compared with DHS fixation.

In a recent biomechanical study, FNS showed significantly higher overall construct stability compared to 3 cannulated screws in an unstable Pauwel III femoral neck fracture^12^. In addition, no significant difference between FNS and DHS was observed regarding biomechanical properties. Although the present study did not investigate the stability of 3 cannulated screws in basicervical neck fracture, it is well documented that DHS or the proximal femoral nail achieves higher fixation strengths than cannulated screws and has been recommended, instead of multiple cannulated screws, for basicervical femoral neck fractures^9,11^. In the present study, there were no significant differences between DHS and FNS in mechanical properties including mean axial stiffness, migration of proximal fragment and screw tip within the femoral head after cyclic load, and failure load. These findings suggest the lack of superiority of any one of the implants between DHS and FNS for the treatment of basicervical femoral neck fracture from a biomechanical point of view. The authors recommend that surgeons select fixation constructs, including DHS and FNS, based on the patient’s anatomy and the surgeon’s comfort with the implant.

Because bone densitometry significantly influences axial stiffness, cycles to neck and leg shortening, cycles to onset of construct failure in DHS, and FNS fixation^12^, it is still unknown whether FNS provides mechanical properties comparable to those of DHS in patients with osteoporosis and basal femoral neck fracture. Rog et al.^21^ reported that DHS constructs with a two-hole side plate were biomechanically comparable with those with four-hole side-plate regard to axial and torsional stiffness and load to failure in an osteoporotic composite femur model with intertrochanteric fracture. Although these results cannot be extrapolated to my comparison (one-hole vs two-hole), FNS with a one-hole side-plate and DHS with a two-hole side-plate showed comparable biomechanical stability in a normal composite femur model with basicervical neck fracture.

Peri-implant fracture distal to the screw hole occurred in 3 cases in the FNS group. However, it is difficult to conclude that FNS is more prone to peri-implant fracture than DHS, as there was no significant difference in the proportion of construct failure between both groups. Peri-implant fracture mostly occurred in different locations between both group: distal to the locking screw hole in the FNS group and around the lag screw in the DHS group. These findings might be explained by more bone loss in the DHS group due to the fixation of the lag screw, the diameter of which is larger than the bolt of FNS. Thus, the smaller diameter of the bolt in FNS might be another advantage from a biomechanical perspective. However, it appears that distal end of locking screw might be stress riser due to short working length in the FNS construct although the risk of peri-implant fracture around locking screw might be reduced by using FNS with two-hole side-plate. Nonetheless, further studies will be required to verify the findings of the present study in an osteoporotic bone model.

The present study has several limitations. The biomechanical study was performed using composite bone, which was made from different materials compared to human bone. However, composite bone models have evolved from rudimentary approximations of human anatomy to high-fidelity, biomechanically relevant replacements for cadaveric bone in many settings^22^. These bones may provide standard sizes and properties between specimens, which will guarantee the reproducibility of the implantation techniques. An artificial fracture to simulate a basicervical femoral neck fracture might not truly reflect the clinical manner in which this fracture develops in vivo. The difference in the screw hole number of side-plate between the two implants might make it difficult to compare them through the biomechanical test directly. However, as a previous study investigated, FNS with a one-hole side-plate is most commonly used as it is minimally invasive^12^. The composite bone used in this study was of normal quality and was filled with solid-density polyurethane foam. Due to the use of this composite bone with normal bone quality, construct failures related to osteoporosis such as cut-out might not have been observed in this study. Several studies reported that screw cut-out risk increased with osteoporosis severity in treating proximal femur fracture, which supports this explanation^23,24^. Accordingly, these results are thought to be applicable to basicervical femoral neck fractures in young adults. Further study will be required to investigate the biomechanical stability of FNS in elderly patients with poor bone quality.

In conclusion, FNS and DHS fixation had comparable biomechanical stability for treating basicervical femoral neck fractures. From a biomechanical point of view, FNS is an acceptable alternative for treating basicervical femoral neck fractures in young adults compared to DHS.

## Methods

### Specimens

**Figure 2:**
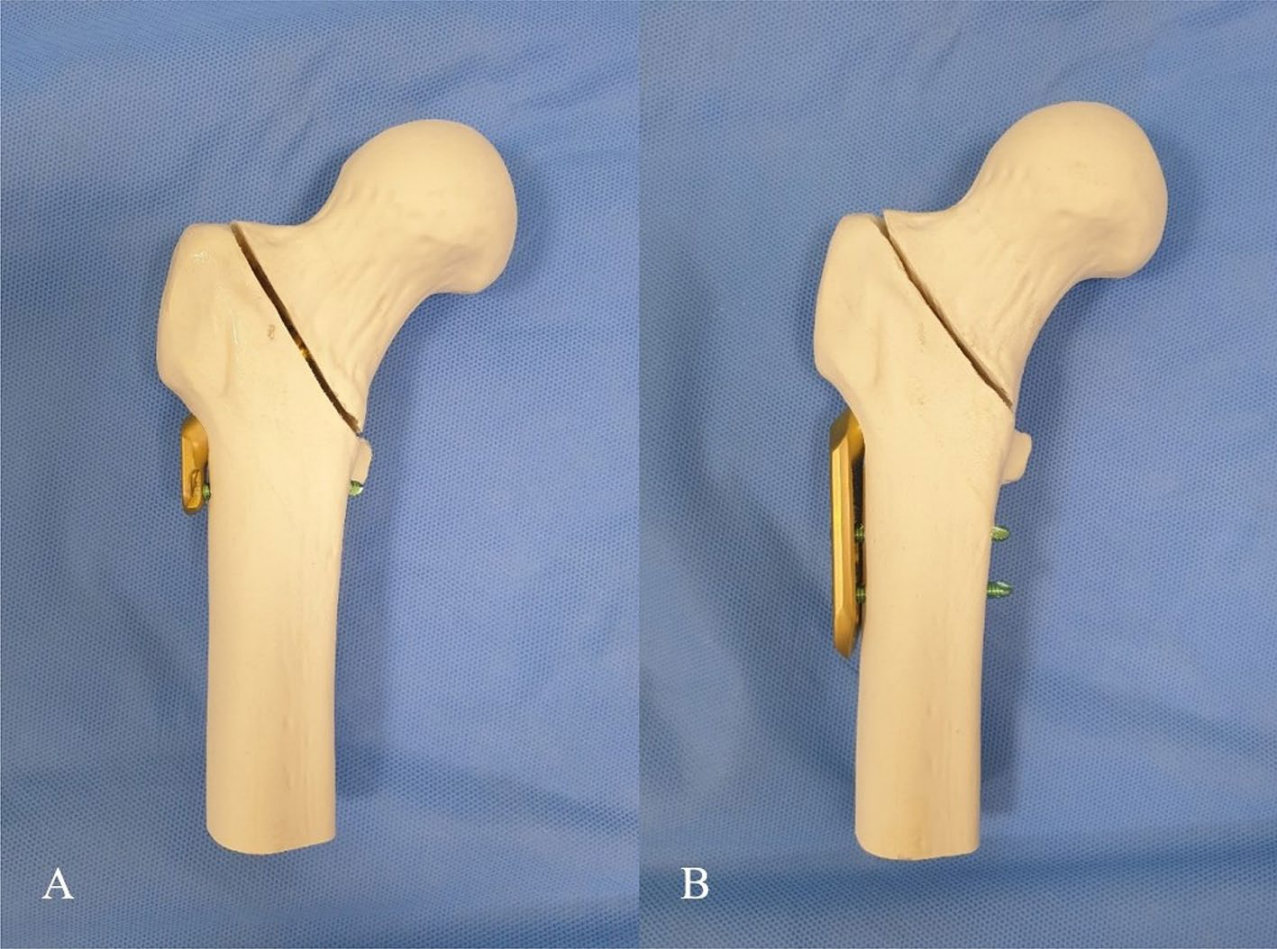
Each specimens in FNS group (**a**) and DHS group (**b**).

Twenty right-sided composite femurs with a customized density (Synbone Sdn 2420, Synbone AG, Switzerland) were used for the present study. These composite femurs were filled with solid density polyurethane foam and coated with a thin cortical layer. Young’s modulus, strength and strain properties of polyurethane-based foam material and adult human femoral bone were described in Table 2^25,26^. The femur models had a length of 337 mm, neck shaft angle of 135°, anteversion of 15°, femoral head diameter of 48 mm, and canal diameter of 10 mm. These specimens were divided into two groups: FNS group (FNS, DePuy Synthes, Zuchwil, Switzerland; n = 10) and DHS group (DHS, DePuy Synthes; n = 10) (Fig. 2).

**Table 2:** Biomechanical properties of polyurethane-based foam material in a composite femur model used in the present study compared with adult human femoral bone.

Parameter	Strain rate (s^−1^)	Value	Strain rate (s^−1^)	Value
Young’s modulus (MPa)	0.001	1180 ± 70	0.5	18,000 ± 2.8
	0.1	1175 ± 73		
0.2% offset yield strength (MPa)	0.001	23 ± 1.22	0.5	84.9 ± 11.2
	0.1	32 ± 2.45		
Strain at 0.2% offset yield strength (%)	0.001	3.6 ± 0.21	0.5	0.62 ± 0.04
	0.1	3.8 ± 0.29		
Ultimate compressive strength (MPa)	0.001	71 ± 2.96	0.5	135.3 ± 34.3
	0.1	66 ± 0.64		
Strain at ultimate compressive strength (%)	0.001	49 ± 1.64	0.5	1.04 ± 0.15
	0.1	47.5 ± 0.45		

*MPa* megapascal.

Basicervical femoral neck fractures corresponding to AO/OTA type 31-B3 were equally produced using an engraving machine based on a drawing. The main fracture line was created at the base of the femoral neck as described by Stafford et al.^27,28^ and the fracture gap was set at 2 mm.

### Surgical technique

**Figure 3:**
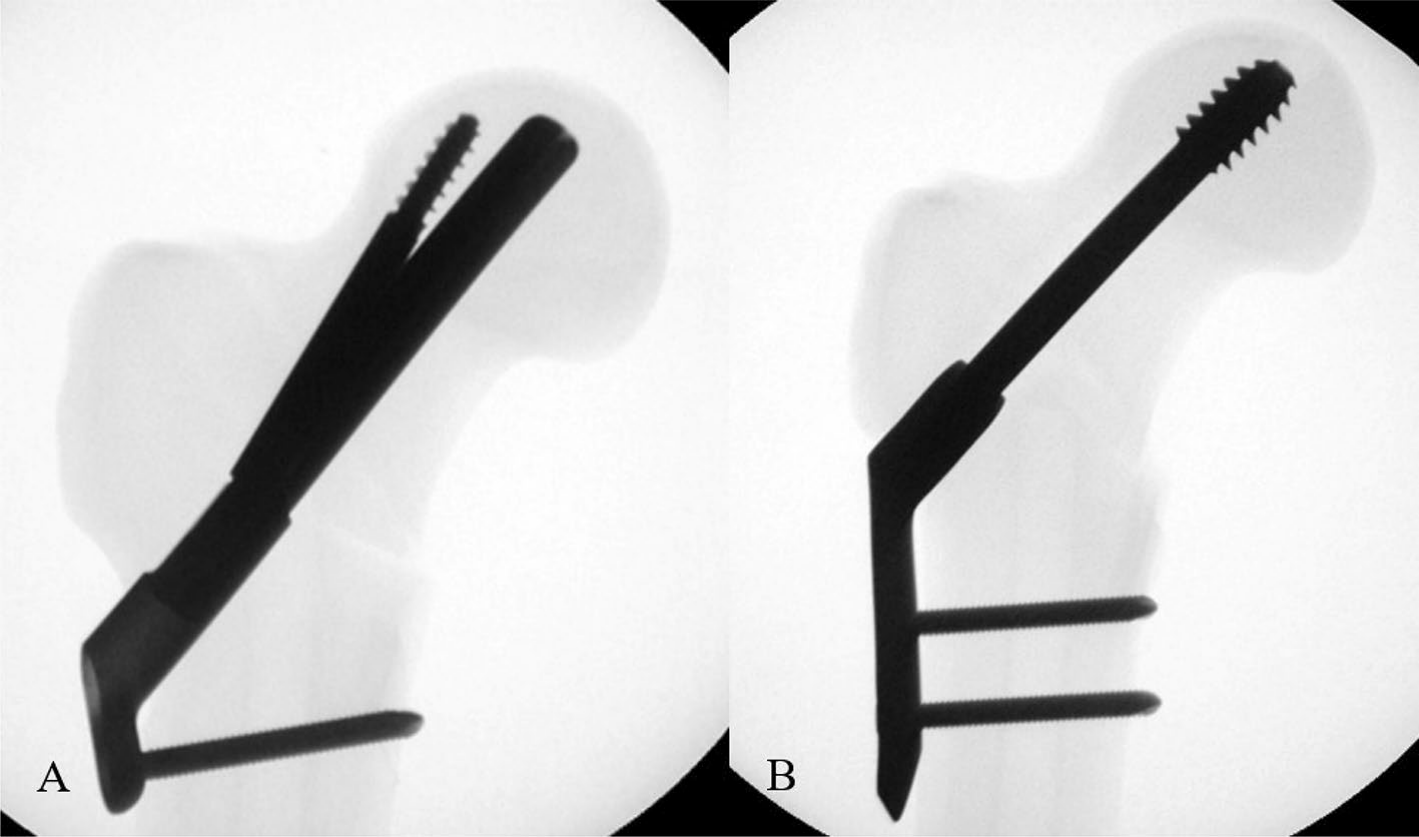
Each specimens in FNS group (**a**) and DHS group (**b**) showed by fluoroscopy.

All specimens were instrumented by the same surgeon with fluoroscopic guidance. A preliminary test using 2 specimens was performed for the exact placement of the screw and bolt in the center-to-center position within the femoral head. The lengths of the screw and bolt were determined to be 100 mm to keep the tip-apex distance less than 20 mm. After a preliminary test, all specimens in the FNS group were fixed with the same one-hole 130° side-plates, bolt and antirotation screw (100 mm), and distal locking screw (46 mm). In the DHS group, all specimens were fixed with the same two-hole 130° side-plates, hip screw (100 mm), and distal locking screws (48 mm). Hip screws or bolts were inserted in the center-to-center position within the femoral head with the approximately same tip-apex distance in all specimens, and this was confirmed with fluoroscopy (Fig. 3).

### Biomechanical test

**Figure 4:**
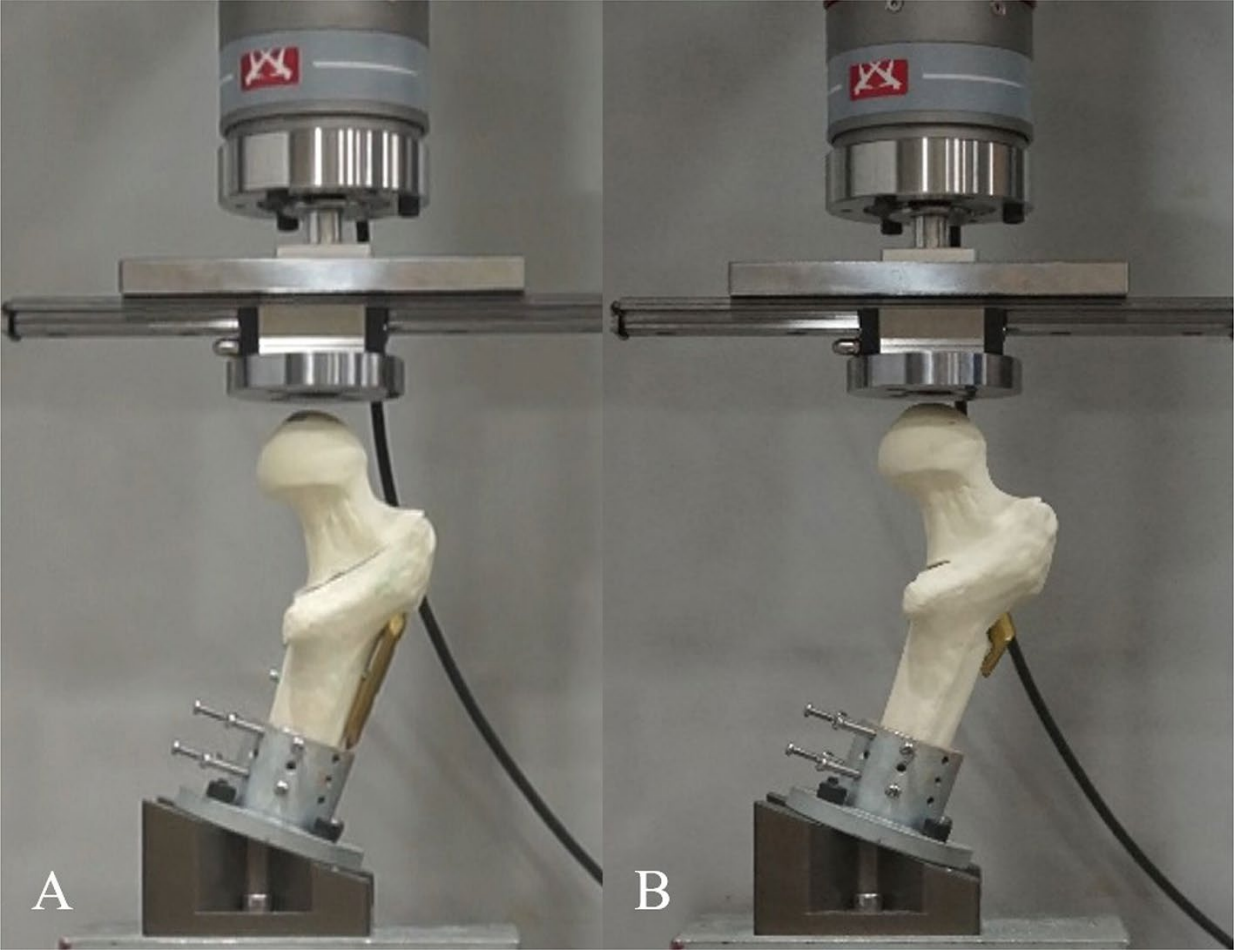
Test setup with a femur specimen instrumentation with (**a**) FNS and (**b**) DHS group.

An Instron ElectroPuls E 3000 All-Electric Dynamic Test Instrument (Instron, Canton, MA, USA) was used for the loading test, with a polished flat applicator that allowed free movement of the femoral head when loaded^29^. Each specimen was tested in 16° adduction of the femoral shaft in accordance with the hip contact forces measured in an in vivo study^30^ (Fig. 4). Three black markers, 1 mm in diameter, were created at the surface of the femoral head so that they could move within the three-dimensional space^31^. Two cameras were placed 60° to the center of the specimen to measure the migration of the proximal fragment according to each axis. Direct linear transformation for three-dimensional coordinates using two cameras was applied to measure the migration and the change in angles in the proximal fragment before and after the experiment^32^.

Two pilot tests were performed to determine the loading protocol on the composite bone models for each implant. The maximum cyclic load was set to 750 N because the femoral shaft fracture occurred distal to the implants at 1500 N. A peak load of 750 N for cyclic loading test was used in several previous biomechanical studies of proximal femur models^33,34^. In addition, to avoid the occurrence of femoral shaft fracture before reaching a peak load, all specimens underwent osteotomy approximately 12 cm proximal from the distal tip^35^.

The loading protocol of each specimen comprised initial quasi-static ramped compression loading from 50 to 200 N at a rate of 15 N/s to ensure complete contact between the femoral head and the flat applicator^11^. Thereafter, cyclic loading was applied with double-peaked physiological vertical loads from 75 to 750 N at a rate of 2 Hz for 10,000 cycles. This number of cyclic loads was considered to simulate the number of steps taken over a postoperative 4- to 6-week period, which was the expected interval for fracture consolidation^36^. After the cyclic loading test, photographs were taken for analysis of the migration and the change in angles in the proximal fragment. A simple radiograph was obtained to investigate the migration of the screw or bolt tip within the femoral head. Thereafter, the peak load, starting at 500 N, was progressively increased until construct failure occurred, while the load–displacement curves were recorded. Construct failure was defined as fracture of the femoral neck, cut-out of screw, implant failure, displacement of fragments more than 15 mm, and a sudden decrease in load resistance observed at the load–displacement curve.

The migration of the proximal fragment was measured using stereophotogrammetry^37^. The rotational change of the proximal fragment before and after cyclic loading was measured using Euler angles^38^, which refer to the angles of rotation of a three-dimensional coordinate frame. After calibration in the laboratory coordinate system based on 3 black markers on the femoral head, the angles of rotation in 3 axes were assessed before and after the experiment^30^.

### Statistical analysis

These results were analyzed using SPSS 18.0 (SPSS, IBM, Armonk, NY, USA). The normal distribution of variables was assessed using the Shapiro–Wilk test. The independent-sample Student’s t-test was used to compare continuous variables including the rotation angles of the proximal fragment, migration of the screw or bolt tip, axial stiffness, and failure load between both groups. Differences in failure type were compared using the chi-square test. Values are represented as the mean and standard deviation. P < 0.05 was considered statistically significant.

An a priori power analysis was undertaken using 0.05 alpha, 1.5 effect size, and 0.8 power, and a minimum sample size of 9 in each group was calculated to detect significant differences in the biomechanical properties between the two groups. An effect size of 1.5 was obtained using estimated mean data from previous biomechanical studies comparing the outcomes after osteosynthesis for proximal femur fracture^11,12,31,35^. A sample size of 10 was chosen in this study.
